# Vulvar contact dermatitis caused by sensitization to colophonium in a patient with type 1 diabetes

**DOI:** 10.1111/cod.13851

**Published:** 2021-05-05

**Authors:** Giuseppina Salzano, Francesca Galletta, Lucia Caminiti, Paolina Lonia, Vittoria Donia, Giovanni B. Pajno, Stefano Passanisi, Fortunato Lombardo

**Affiliations:** ^1^ Department of Human Pathology in Adult and Developmental Age "Gaetano Barresi" University of Messina Messina Italy

**Keywords:** case report, diapers, glucose sensor, patch test, resin, sanitary pads

Colophonium is a natural product consisting of a complex mixture of more than 100 substances derived from various species of standing pine trees.^1^ It can be used in both unmodified and modified forms as a fast‐acting adhesive for commercial, medical, and industrial products. Unmodified colophonium consists of about 90% resin acids. The remaining 10% is known as the neutral fraction and consists of terpenes, terpene alcohols, sesquiterpenes and diterpene hydrocarbons, aldehydes, and alcohols.^2^ Colophonium is a well‐known sensitizer and its main allergens are 222 products of unmodified and modified colophonium and some of the new resin acids, synthesized during modification. Recently, colophonium has been increasingly associated with allergic contact dermatitis (ACD) caused by medical devices used for the management of diabetes.^3^, ^4^, ^5^

## CASE REPORT

**FIGURE 1:**
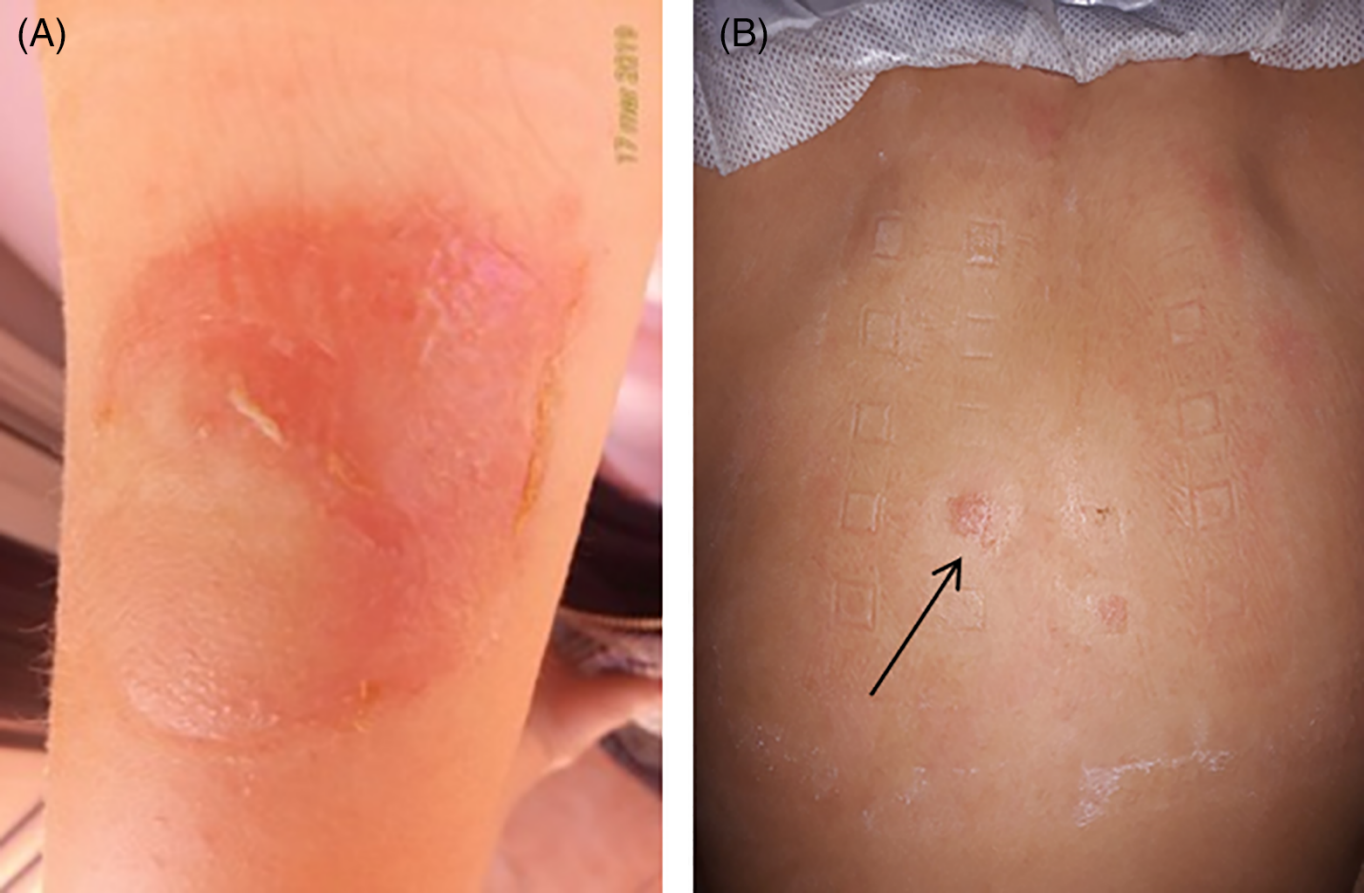
(**A**) Erythematous lesion associated with vesicles at the site of insertion of glucose sensor and (**B**) patch testing results showing a strong positive reaction to colophonium (black arrow)

We report a case of a 13‐year‐old, non‐atopic girl with recurrent flares of ACD caused by colophonium. She has suffered from type 1 diabetes (T1D) since the age of 7 years and had been treated with multiple daily injections up to the age of 9. She was then changed to continuous subcutaneous insulin infusion therapy because of brittle glycemic control. At the same time, a continuous glucose monitoring (CGM) device (Enlite sensor, Minneapolis, Minnesota) was introduced to intensify her glycemic level monitoring. After 2 years, she developed erythema, papules, and vesicles on her arms at the site of insertion of the CGM device, suggesting ACD (Figure 1A). She was patch tested with specific allergens belonging to resin and acrylate classes (butyl acrylate 0.1%, butanediol 1–3 methacrylate 2%, colophonium 20%, ethyl acrylate 0.1%, isobornyl acrylate 0.1%, methyl methacrylate 2%, para tertiary butylphenol formaldehyde resin 1%, phenol formaldehyde resin 5%, sesquiterpene 0.1%, toluenesulfonamide formaldehyde resin 10%, and urea formaldehyde resin 10%). Petrolatum (pet.) was used as the vehicle. Patch tests (Allergopharma, Reinbek, Germany) were performed with 8 mm Finn Chambers (SmartPractice, Phoenix, Arizona) on Scampor Tape (Norgerplaster, Vennesla, Norway). Test chambers containing allergens were placed on the skin of the back and were removed after 48 hours. Patch tests were read 30 minutes after removal on day (D) 2 and 1 day later (D3). Readings on D3 resulted in a strong positive reaction (++) to colophonium 20% pet. and a weak reaction (+) to butanediol 1–3 methacrylate 2% pet. (Figure 1B). Treatment with methylprednisolone ointment and emollient was initiated and the dermatitis rapidly cleared within a few days. The presence of colophonium in the adhesive of the glucose sensor was confirmed by the manufacturer of the device.

At the age of 12, the patient experienced menarche and started to use common sanitary pads. After 6 days from the first use, she presented with itchy rash affecting the vulvar area. Although the patient recovered from these symptoms with the use of topical emollient creams and cleaners, vulvar lesions occurred at each menstruation. Based on her past history of sensitization to colophonium, manufacturers of the product were contacted and they stated that colophonium was present in the sanitary pads in minimal amounts (ie, <1%). Thus, the patient started using 100% cotton tampons and no further lesions occurred.

## DISCUSSION

Allergic contact dermatitis is an important, but often overlooked, cause of vulvar disorder. Vulvar ACD was first reported in 1990^6^ and the main documented allergens are medicaments (e.g. local anesthetics and corticosteroids), fragrances, and preservatives.^7^ Cases of vulvar ACD caused by sensitization to colophonium have already been described.^8^ Indeed, colophonium has been identified among the ingredients of several sanitary pads and diapers.^9^ According to EU legislation (EC No. 1272/2008) products containing at least 1% colophonium/rosin must be classified and labelled as “H317: May cause an allergic skin reaction”.^5^ Vulvar tissue is more permeable than skin due to differences in structure, occlusion, hydration, and susceptibility to friction.^10^ Therefore, a low eliciting dose of allergen(s) can lead to the appearance of vulvar ACD. Our patient was highly sensitized to colophonium, as demonstrated by her strongly positive patch test reactions, due to her prolonged use of a CGM device.

Vulvar ACD is not easy to diagnose in the pediatric age as female adolescents are often reluctant to declare their symptoms both to parents and doctors. Therefore, pediatricians should be aware of the risk of ACD induced by sanitary pads and should recommend cotton tampons or bamboo fiber pads to girls with an ascertained sensitization to colophonium.

## CONFLICT OF INTEREST

The authors declare no conflicts of interest.

## AUTHOR CONTRIBUTIONS

**Giuseppina Salzano:** Writing‐original draft. **Francesca Galletta:** Writing‐original draft. **Lucia Caminiti:** Data curation. **Paolina Lonia:** Data curation. **Vittoria Donia:** Data curation. **Giovanni Pajno:** Writing‐review & editing. **Stefano Passanisi:** Writing‐review & editing. **Fortunato Lombardo:** Conceptualization; writing‐review & editing.
